# Enhanced protopanaxadiol production from xylose by engineered *Yarrowia lipolytica*

**DOI:** 10.1186/s12934-019-1136-7

**Published:** 2019-05-18

**Authors:** Yufen Wu, Shuo Xu, Xiao Gao, Man Li, Dashuai Li, Wenyu Lu

**Affiliations:** 1 School of Chemical Engineering and Technology, Tianjin University, Tianjin, People’s Republic of China; 2 Key Laboratory of System Bioengineering (Tianjin University), Ministry of Education, Tianjin, People’s Republic of China; 3 SynBio Research Platform, Collaborative Innovation Center of Chemical Science and Engineering (Tianjin), Tianjin, People’s Republic of China

**Keywords:** Protopanaxadiol, Xylose, *Yarrowia lipolytica*, Metabolic engineering, Synthetic biology

## Abstract

**Background:**

As renewable biomass, lignocellulose remains one of the major choices for most countries in tackling global energy shortage and environment pollution. Efficient utilization of xylose, an important monosaccharide in lignocellulose, is essential for the production of high-value compounds, such as ethanol, lipids, and isoprenoids. Protopanaxadiol (PPD), a kind of isoprenoids, has important medical values and great market potential.

**Results:**

The engineered protopanaxadiol-producing *Yarrowia lipolytica* strain, which can use xylose as the sole carbon source, was constructed by introducing xylose reductase (XR) and xylitol dehydrogenase (XDH) from *Scheffersomyces stipitis*, overexpressing endogenous xylulose kinase (ylXKS) and heterologous PPD synthetic modules, and then 18.18 mg/L of PPD was obtained. Metabolic engineering strategies such as regulating cofactor balance, enhancing precursor flux, and improving xylose metabolism rate via XR (K270R/N272D) mutation, the overexpression of tHMG1/ERG9/ERG20 and transaldolase (TAL)/transketolase (TKL)/xylose transporter (TX), were implemented to enhance PPD production. The final Y14 strain exhibited the greatest PPD titer from xylose by fed-batch fermentation in a 5-L fermenter, reaching 300.63 mg/L [yield, 2.505 mg/g (sugar); productivity, 2.505 mg/L/h], which was significantly higher than the titer of glucose fermentation [titer, 167.17 mg/L; yield, 1.194 mg/g (sugar); productivity, 1.548 mg/L/h].

**Conclusion:**

The results showed that xylose was more suitable for PPD synthesis than glucose due to the enhanced carbon flux towards acetyl-CoA, the precursor for PPD biosynthetic pathway. This is the first report to produce PPD in *Y. lipolytica* with xylose as the sole carbon source, which developed a promising strategy for the efficient production of high-value triterpenoid compounds.

**Electronic supplementary material:**

The online version of this article (10.1186/s12934-019-1136-7) contains supplementary material, which is available to authorized users.

## Background

Lignocellulose from wood in forestry and agriculture as well as industrial waste can reach 100 billion tons/year, making this biomass the most abundant renewable resource on the Earth [1]. Efficient utilization of lignocellulose is essential for reducing demands for energy and food. Xylose is the second most abundant monosaccharide in lignocellulosic hydrolysate following glucose, accounting for nearly 35% of all monosaccharides [2]. However, most microorganisms cannot efficiently metabolize xylose from lignocellulosic hydrolysate due to the carbon catabolite repression effect [3], which largely limits the applications of lignocellulose. Thus, the use of xylose has become a hot issue in the study of lignocellulose.

In recent years, great progress has been made in xylose metabolism studies. Various compounds have been successfully obtained via microbial metabolism of xylose, such as xylitol [4], ethanol [5], acetoin [6], fumaric acid [7], and polyhydroxyalkanoate [8]. As a model organism for lipid metabolism, *Yarrowia lipolytica* does not naturally metabolize xylose, primarily due to low expression levels of the key enzymes involved in the xylose metabolic pathway [9–12]. However, whether the *Y. lipolytica* strain can grow using xylose as a substrate remains controversial [13–15]. There have been many attempts to engineer Y. lipolytica to use xylose as a substrate. Ledesma-Amaro et al. [16] have engineered *Y. lipolytica* to metabolize xylose to produce lipids and citric acid by overexpression of xylose reductase (XR) and xylitol dehydrogenase (XDH) from *Scheffersomyces stipitis* and endogenous xylulose kinase (ylXKS). The growth ability of engineered *Y. lipolytica* using xylose was identical to that of the wild type strain grown using glucose. This mutant could produce up to 80 g/L of citric acid from xylose. Li and Alper [17] have implemented a starvation adaptation strategy to improve the metabolic rate of xylose. They introduced a heterologous oxidoreductase pathway to optimize xylose utilization by *Y. lipolytica* in a stable manner; this mutant produced > 15 g/L of lipid via bioreactor fermentation, with a maximal lipid productivity of 0.19 g/L/h.

*Yarrowia lipolytica* contains the native mevalonate (MVA) pathway to provide precursor compounds, namely isopentenyl pyrophosphate and dimethylallyl pyrophosphate [18], indicating that *Y. lipolytica* can serve as a natural host for terpenoid synthesis. *Y. lipolytica* is a potential platform for producing isoprenoids using acetyl-CoA as the precursor due to its convenient genetic manipulation, robust acetyl-CoA synthesis, NADPH, and energy supply system [19–22], as *S. cerevisiae* lacks acetyl-CoA [23, 24]. In previous studies, biosynthesis of many terpenoids were realized in *Y. lipolytica*, such as farnesene, limonene, and ginsenoside compound K [25–27], and engineering strategies have been employed to enhance the production, such as codon optimization, heterologous synthetic genes introduction, synthetic pathway up-regulation, and competitive pathway down-regulation. Limonene (23.56 mg/L; 1.36 mg/g DCW) was obtained in *Y. lipolytica* by codon optimization and overexpression *HMG1* and *ERG12* genes [25]; The α-farnesene titer was increased by 20.8-fold by *tHMG1*, *IDI*, and *ERG20* overexpression, reaching 259.98 mg/L [26]; The titer of ginsenoside compound K was increased to 161.8 mg/L by a combination of metabolic engineering strategies [27].

Protopanaxadiol (PPD) is a natural C_30_ isoprenoid with important medical applications owing to its anticancer, antitumor, antiviral, and antibiotic properties [28–30]. The extraction method for PPD from plants has limited applications because of shortage of ginseng plants. Therefore, strategies have been developed for PPD biosynthesis to overcome the limitations of traditional extraction processes [31, 32].

**Fig. 1:**
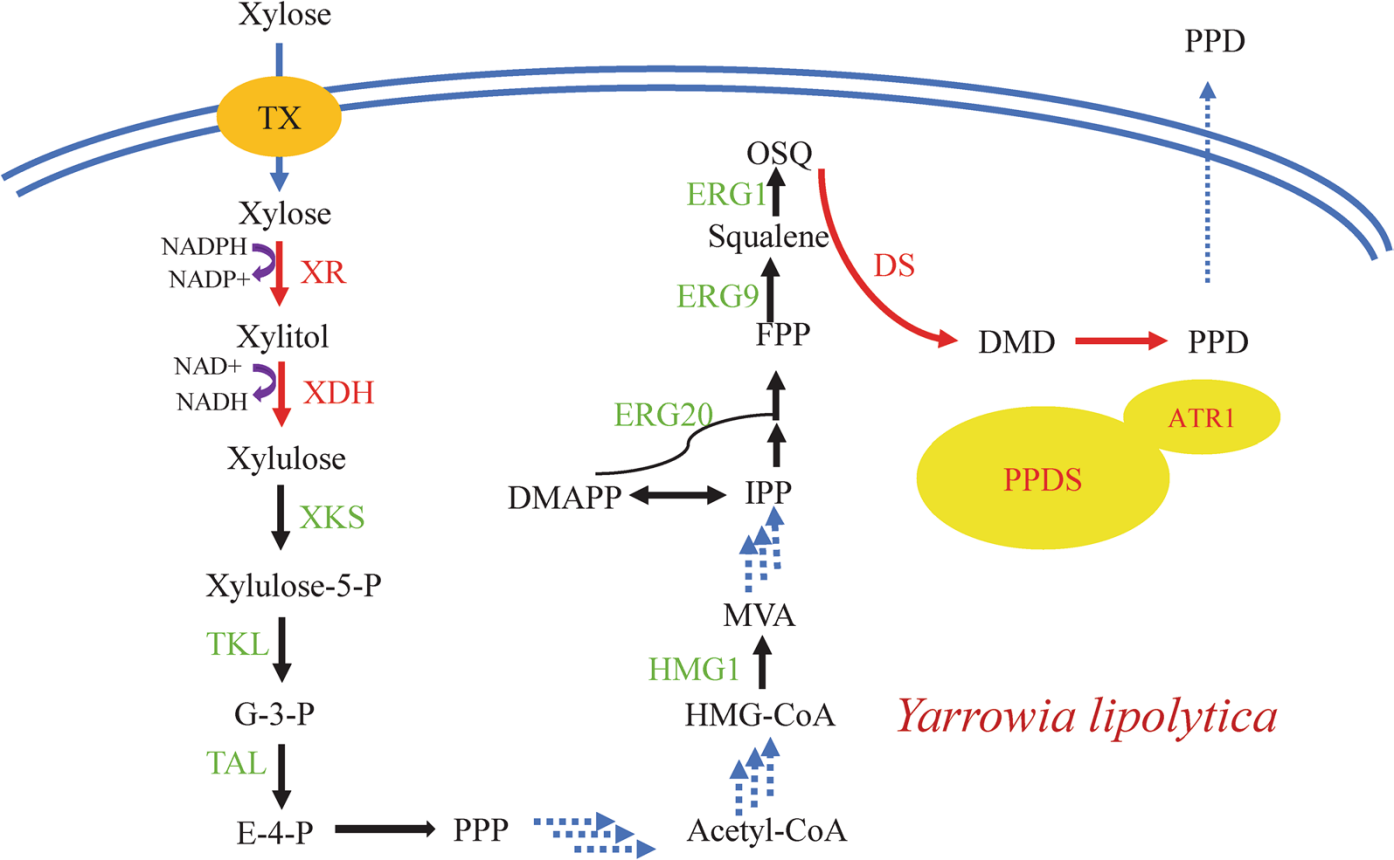
Metabolic engineering strategies for producing PPD from xylose. TX, sugar transporter; XR, xylose reductase; XDH, xylitol dehydrogenase; XKS, Xylulose kinase; TKL, transketolase; TAL, transaldolase; HMG1, 3-hydroxy-3-methylglutaryl-coenzyme A reductase; ERG9, squalene synthase; ERG20, farnesyl pyrophosphate synthase; ERG1, squalene monooxygenase; DS, DMD synthase; PPDS, PPD synthase; ATR1, NADPH-P450 reductase; G-3-P, glyceraldehyde-3-phosphate; E-4-P, erythrose-4-phosphate; PPP, pentose phosphate pathway; HMG-CoA, hydroxymethylglutaryl-CoA; MVA, mevalonate acid; OSQ, 2,3-oxidized squalene; DMD, dammarenediol-II; PPD, protopanaxadiol. Green represents the overexpressed native genes and red represents the optimized heterologous genes. Three dotted arrows represent multi-step reactions

Xylose fermentation is more desirable for isoprenoid production than glucose fermentation by *Saccharomyces cerevisiae* due to a rigid flux partition toward ethanol during glucose metabolism [33–35]. However, there are challenges that must be overcome during isoprenoid production from xylose in *S. cerevisiae*, such as cofactor imbalance, slow xylose metabolism rate, and insufficient acetyl-CoA supply. In this study, *Y. lipolytica* was selected as the host for PPD production from xylose due to robust acetyl-CoA synthesis, NADPH, and energy supply system. Engineered *Y. lipolytica* strains that could metabolize xylose were constructed to evaluate the metabolic capacity of xylose by introducing XR and XDH from *S. stipitis* or from *Y. lipolytica*. PPD production was enhanced by overexpressing specific components of the MVA pathway, enhancing the xylose transport rate, and strengthening the metabolic pathway (Fig. 1). Fermentation was performed using xylose, glucose, and mixed sugar as carbon sources to evaluate the performance of different substrates to produce PPD titers. Finally, fermentation amplification was conducted in a 5-L bioreactor to examine the stability xylose metabolism for PPD production.

## Results and discussion

### *Ku70* knockout to enhance homologous recombination efficiency of *Y. lipolytica*

**Fig. 2:**
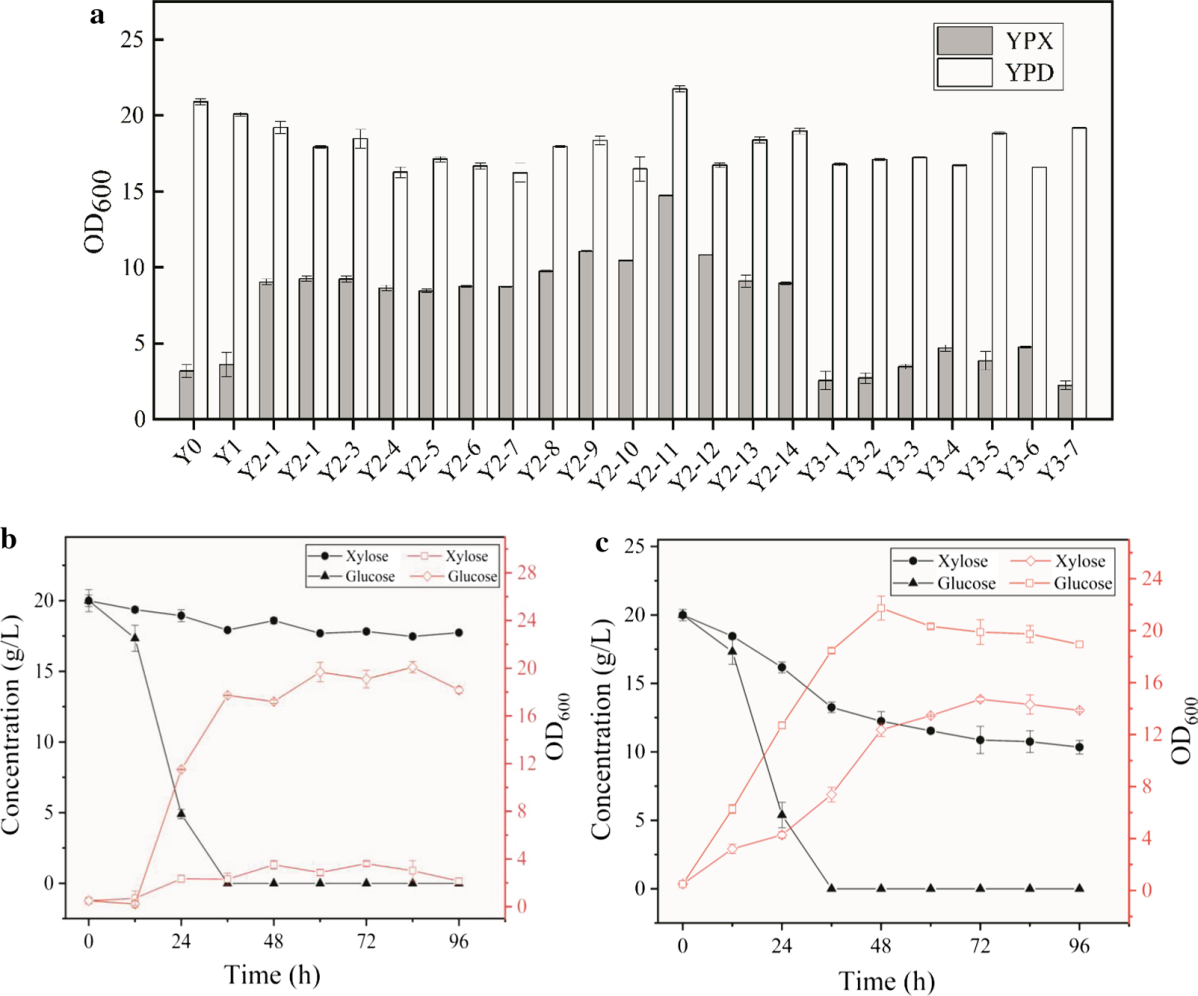
The growth of different engineered strains from xylose and glucose, respectively. **a** The OD_600_ of Y0 (*Yarrowia lipolytica* ATCC 201249), Y1 (*Ku*70 deletion), Y2-1_Y2-14 (introducing heterologous xylose metabolic pathway) and Y3-1_Y3-7 (introducing endogenous metabolic pathway) in YPD and YPX medium, respectively. **b** The OD_600_ and sugar concentration curves of Y1 from xylose and glucose as substrates, respectively. **c** The OD_600_ and sugar concentration curves of Y2 from xylose and glucose as substrates, respectively. Each data represents an average ± 1 standard deviation of three parallel fermentations

*Yarrowia lipolytica* is a promising industrial producer of lipids due to its fully annotated genome, ease of manipulation, and ability to utilize hydrophobic carbon sources as substrates [36, 37]. However, homologous integration of exogenous DNA can be difficult because *Y. lipolytica* mainly prefers the non-homologous end-joining (NHEJ) recombination rather than the homologous recombination (HR) [38]. Lustig [39] has confirmed that both KU70 and KU80 bind to broken DNA ends exhibit bridging activity regardless of the sequence homology of the broken ends. To increase the rate of HR in *Y. lipolytica*, *Ku70* deletion cassettes with the LoxP–URA3–LoxP marker were generated to block the NHEJ pathway and were transformed into *Y. lipolytica* ATCC 201249 to obtain the Y1 strain. HR efficiency increased from 28 to 54% after *Ku70* knockout. Shake flask fermentation (Fig. 2a) revealed no difference in cell growth, indicating that *Ku70* knockout had a great effect in improving the HR efficiency of *Y. lipolytica* and did not adversely affect cell growth, which is important for the construction of engineered strains. In previous studies, the HR frequency is also dramatically increased in *Y. lipolytica* by disrupting the *ku70* gene [40, 41]. However, *Ku70* knockout strain may have higher instability than wild type. The alternative approaches to transiently downregulate *ku70* can be considered to improve gene integration, which have been confirmed in *Trichoderma reesei* [42].

### Introduction of the xylose metabolic pathway in *Y. lipolytica*

The natural capacity of *Y. lipolytica* to metabolize xylose remains unclear. To obtain xylose-fermenting strains, endogenous and heterologous pathways for xylose metabolism were introduced into *Y. lipolytica*. The cofactor imbalance problem can be resolved using mutated XR [43–45]. Watanabe et al. [43] have induced two XR gene mutations, R276H and K270R/N272D, resulting in change in preference from NADPH to NADH, which led to respectively 52- and 146-fold increases in catalytic efficiency of the mutant strains compared with that of the original strain. *XYL1* and *XYL2* genes from *S. stipitis* encoding XR (K270R/N272D) and XDH, respectively, were codon-optimized according to codon preference of *Y. lipolytica* and transformed into the Y1 strain, together with the *ylXKS* expression cassette, to produce the strain Y2 (Fig. 1). Moreover, the endogenous *ylXYL1*, *ylXYL2*, and *ylXKS* genes were integrated into the *zeta* site of *Y. lipolytica* using the same promoters as those of the heterologous genes to obtain the Y3 strain. *Y. lipolytica* ATCC 201249 could not grow in xylose medium (Fig. 2a). The Y2 series of engineered strains with the introduced heterologous xylose metabolic pathway showed a significant increase in OD_600_ values compared with the original strain, indicating that XR and XDH derived from *S. stipitis* were successfully expressed in *Y. lipolytica*. However, the Y3 strain overexpressing the endogenous xylose metabolism genes showed no obvious growth, indicating that endogenous XR and XDH cannot use xylose as the sole carbon source.

Strain with the highest OD_600_ using xylose was selected for shake flask fermentation, with Y0 and Y1 as comparisons. Results indicated that the Y2 strain alone consumed approximately 10 g/L xylose and that the growth ability using xylose remained lower than that using glucose (Fig. 2b, c). Therefore, we speculated that improving the efficiency of sugar transporters [46–48] or enhancing the adaptability of strains to xylose [49] can enhance xylose utilization.

### Adaptation to improve xylose metabolism

**Fig. 3:**
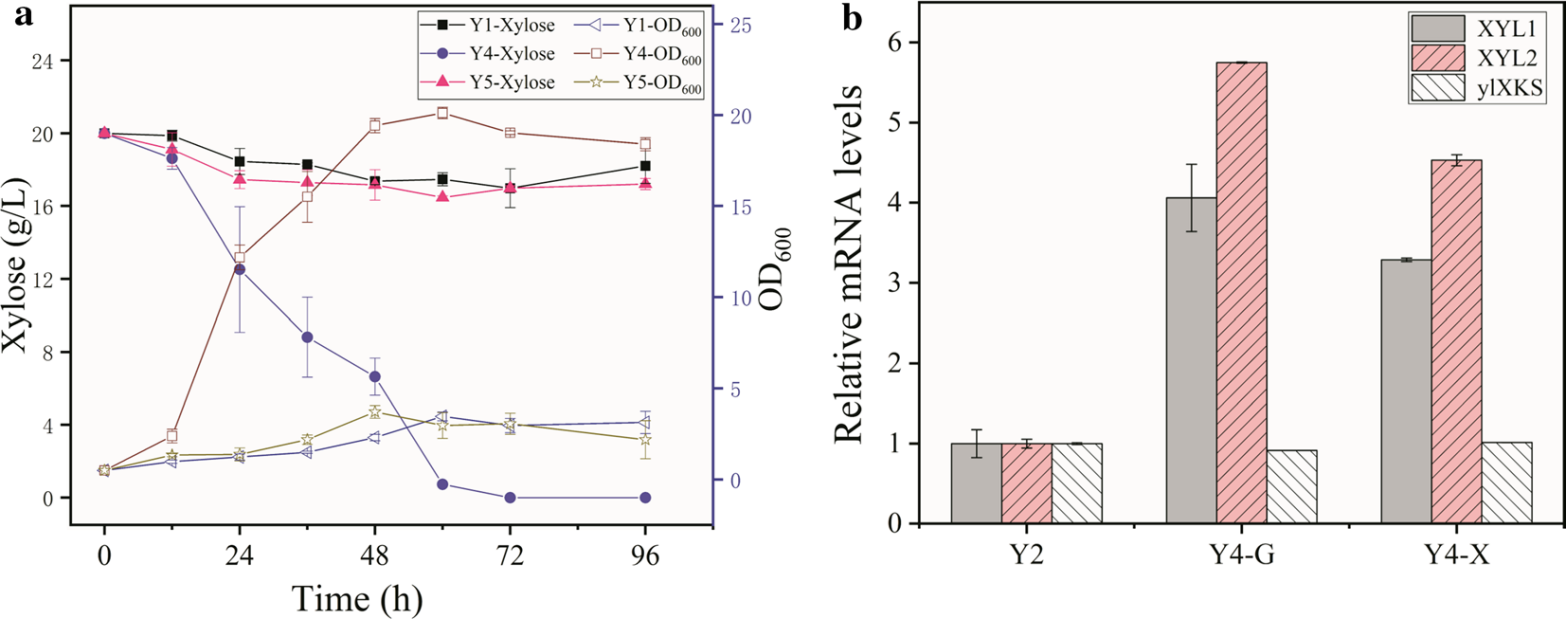
The effects of different strains after adaptation. **a** The OD_600_ and sugar concentration curves of Y4 and Y5 by adaptation of Y2 and Y3, respectively, compared with Y1. **b** Relative mRNA expression levels of *XYL1*, *XYL2* and *ylXKS* at exponential phase for strain Y2 and Y4, were measured with RT-qPCR. Y2 were grown on glucose. Y4 was grown on either glucose and xylose. The relative mRNA levels of the strain Y2 were 1. Each data represents an average ± 1 standard deviation of three parallel fermentations

Studies have reported that *Y. lipolytica*, with introduced heterologous xylose metabolic pathway, can utilize xylose, although the phenotype of xylose catabolism is unstable [50]. Therefore, we used the adaption strategy to overcome the instability of *Y. lipolytica* growth in xylose. The strains Y2 and Y3 were cultured repeatedly in SC medium containing xylose for a long time, and obtained strains Y4 and Y5, respectively. The Y5 strain showed no significant increase in OD_600_ compared with the Y1 strain, whereas the OD_600_ of the Y4 strain reached 20.12 (Fig. 3a). In addition, Y4 consumed 20 g/L xylose in 72 h, and the rate of xylose metabolism increased 1.69-folds the rate by Y2. RT-qPCR was performed to analyze the difference in the rate of xylose metabolism after adaptation. We observed that the expression levels of exogenous *XYL1* and *XYL2* in Y4 were indeed much higher than the strain Y2 (Fig. 3b), which would be beneficial for promoting xylose metabolism. We also measured the expression level of *ylXKS* under the same conditions, and observed no substantial changes of *ylXKS* expression between the strains (Fig. 3b). These results indicated that adaptation to xylose could significantly promote xylose utilization and improve the stability of *Y. lipolytica* in xylose, which was probably due to the improvement of *XYL1* and *XYL2* expression levels after adaptation.

### PPD production in xylose-metabolizing strains of *Y. lipolytica*

**Fig. 4:**
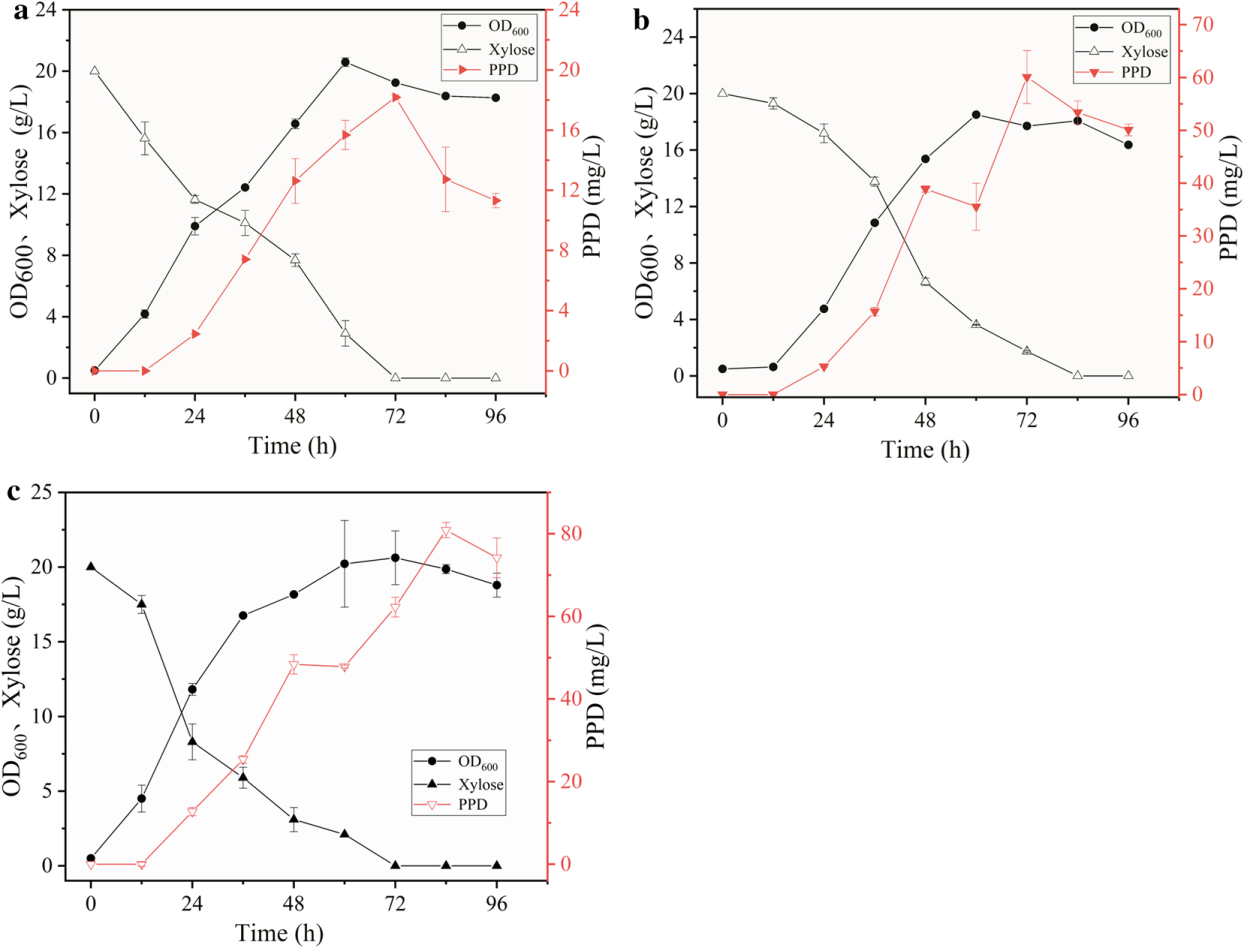
The production of PPD in different strains utilizing xylose. **a** The production of PPD in strain Y7 by co-expression of PPD synthesis modules. **b** The production of PPD in strain Y8 by fusion expression PPD synthesis modules. **c** Fermentation of strain Y10 overexpressing MVA pathway for enhancing PPD production at shake flask level. Each data represents an average ± 1 standard deviation of three parallel fermentations

The *leu2* and *ura3* markers were used in the construction of engineered strain Y4. To recycle the marker gene *ura3*, the CRE-expressing plasmid pINA1269-CRE was used, resulting in the Y6 strain. The PPD synthesis pathway including dammarenediol-II (DS) synthase, cytochrome P450 monooxygenase (PPDS), and NADPH-P450 reductase (ATR1), has previously been successfully expressed in *Y. lipolytica* [27]. In this study, co-expression and fusion expression modules (Additional file 1: Figure S1) for PPD synthesis were integrated into the *Y. lipolytica* Y6 genome, as previously described, resulting in the Y7 and Y8 strains, respectively. The Y7 and Y8 strains performed fermentation in YPX medium while shaking (Fig. 4a, b). In YPX medium, PPD titer from strain Y8 was 60.1 mg/L, and increased 2.3 times compared with Y7 (18.18 mg/L). PPD yield from Y8 by fusion expression was significantly higher than that from Y7 by co-expression, indicating that the difference in PPD yield between the two strains was due to expression of the PPDS and ATR1. Increasing the precursor supply may further increase PPD production.

### Overexpression of the MVA pathway to enhance PPD production

The MVA pathway is the only source of precursors for the synthesis of terpenoids in *Y. lipolytica* [51–53]. Several key enzymes in the MVA pathway have been investigated to increase PPD production in *S. cerevisiae* or *Y. lipolytica*, such as tHMG1, ERG10, ERG12, ERG9, ERG20, and ERG13 [32, 35]. The key enzymes, tHMG1, ERG9, and ERG20, were overexpressed in Y9 (the *ura3* marker in Y8 was recycled), resulting in the Y10 strain, in which PPD titer increased by 34% compared with that in the Y8 strain, reaching 80.88 mg/L, although OD_600_ of the cells did not change significantly (Fig. 4c). Overexpression of the MVA pathway significantly increased PPD production; however, in *Y. lipolytica*, PPD yield from xylose could be further increased by increasing the metabolic rate of xylose.

### Optimization of the xylose assimilation pathway to increase the xylose metabolism rate

**Fig. 5:**
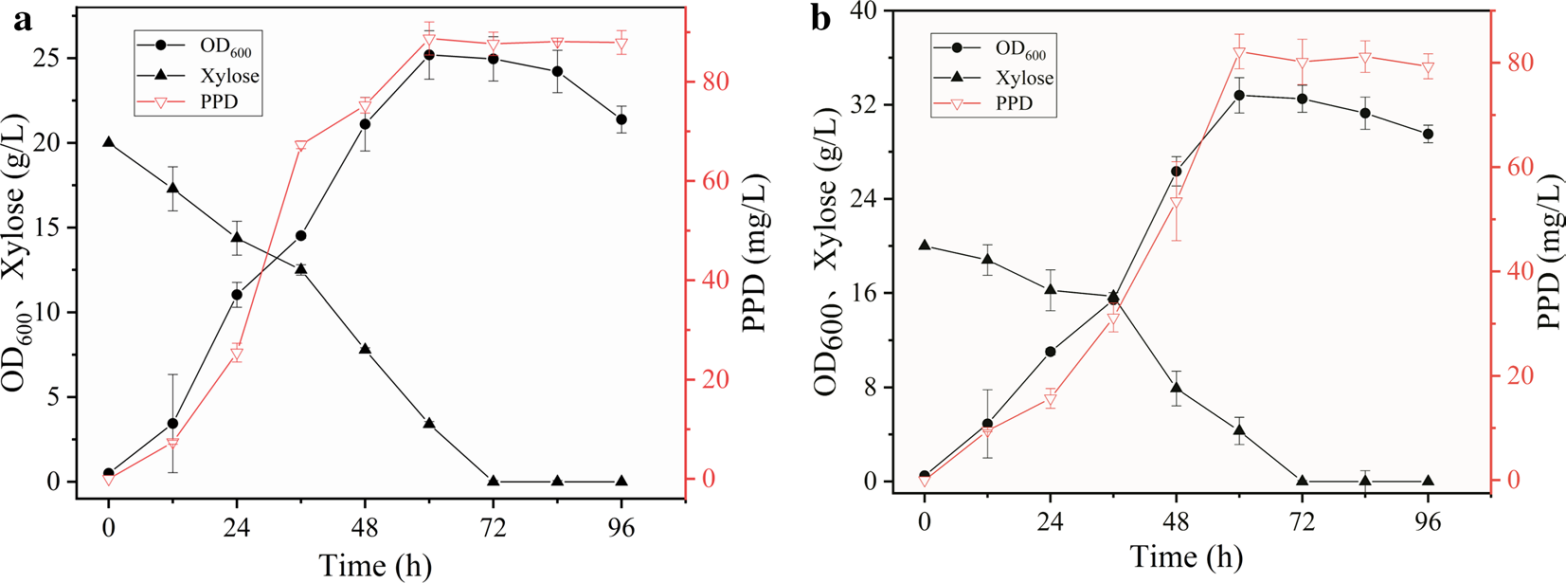
Shake flask fermentation of engineered strains that enhanced the xylose metabolic pathway for PPD production. **a** Fermentation of strains Y12 overexpressing TKL and TAL for improving PPD production from xylose. **b** Fermentation of strains Y14 overexpressing TX for improving PPD production from xylose. Each data represents an average ± 1 standard deviation of three parallel fermentations

Optimization of the xylose assimilation pathway involves overexpression of xylose transporters to increase the xylose transport rate and improve the flux of xylulose into the pentose phosphate pathway. The xylose transporter in *Y. lipolytica* is in a dormant state; however, the xylose assimilation rate was enhanced by approximately 50% by overexpressing xylose transporters (YALI0B00396p) [54]. Meanwhile, acetyl-CoA is a basic precursor for terpenoid synthesis. Xylose needs to enter the central carbon metabolism via the pentose phosphate pathway, through which acetyl-CoA is derived. Transaldolase (TAL) and transketolase (TKL)—the key enzymes entering the pentose phosphate pathway—are immediately downstream of the xylose degradation pathway. TAL and TKL have been successfully expressed in *S. cerevisiae* to promote xylose metabolism [35, 55]. In the present study, the *ura3* marker in the Y10 strain was recycled to obtain the Y11 strain. Endogenous *TAL* and *TKL* genes were overexpressed in the Y11 strain via HR, resulting in the Y12 strain. The *ura3* marker in Y12 was recycled to obtain the Y13 strain. The Y14 strain was obtained from the Y13 strain by introducing TX (YALI0B00396p) expression modules. Fermentation results are summarized in Fig. 5. OD_600_ of the Y12 strain increased to 25.03, indicating that the biomass improved as a result of overexpression of *TAL* and *TKL*. In addition, PPD titer increased to 88.73 mg/L, indicating that the flux of xylose to the central pentose phosphate pathway had increased. The overexpression of xylose transporter (TX) decreased PPD yield (about 80 mg/L), however, OD_600_ increased significantly (32.81), indicating that the metabolic rate of xylose had increased and that more carbon source was converted to biomass.

### Shake flask fermentation of the engineered strain Y14 using xylose to produce PPD

Mixed sugar fermentation is often used as an effective strategy to improve the comprehensive utilization of sugars [50, 56, 57]. To explore the feasibility of mixed sugar fermentation by *Y. lipolytica*, optimization of the initial sugar concentration and fermentation of mixed sugar were performed using the engineered Y14 strain. The Y14 strain was allowed to ferment initial sugar concentrations of 20, 30, 40, 50, 60, 70, and 80 g/L, respectively, in YPD and YPX media for 4 days. OD_600_ at a concentration of 60 g/L xylose was the maximum, reaching 34.3 (Table 1). The initial concentration of xylose consumed by the Y14 strain was 40 g/L, and PPD yield using 40 g/L xylose was significantly higher than that using low sugar concentrations. In addition, at different sugar concentrations, PPD titer using xylose was higher than that using glucose, indicating that the biomass and PPD production by the Y14 strain using xylose were better than those using glucose. In terms of biomass and PPD production, 40 g/L was selected as the initial optimal sugar concentration for fermentation of xylose and glucose by the Y14 strain.

**Table 1:** Fermentation of Y14 at different initial sugar concentrations

Initial sugar concentration (g/L)	Symbol^a^	OD_600_	Residual sugar concentration (g/L)^b^	PPD titer (mg/L)^b^
20	G	21.7	0	57.17 ± 1.83
	X	26.8	0	81.23 ± 1.47
30	G	29.6	0	61.17 ± 1.31
	X	24.1	0	80.45 ± 0.71
40	G	27.4	0	65.27 ± 1.28
	X	30.5	0	120.36 ± 1.59
50	G	30.3	0	58.62 ± 2.22
	X	32.8	1.0 ± 0.1	124.94 ± 2.75
60	G	31.4	0	87.85 ± 1.57
	X	34.3	12.1 ± 0.9	128.94 ± 0.43
70	G	33.4	2.2 ± 0.4	82.27 ± 2.31
	X	30.4	17.9 ± 0.8	118.55 ± 4.42
80	G	32.7	12.9 ± 0.6	80.45 ± 1.74
	X	29.9	26.3 ± 1.2	135.21 ± 2.76

^a^Represents the type of sugars. G and X represent glucose and xylose, respectively

^b^Each data represents an average ± 1 standard deviation of three parallel fermentations

To explore effects of mixed sugar on PPD production, mixed sugars and individual sugars were used for fermentation by Y14. Total concentration of sugar was set to 40 g/L, and the proportions of xylose used were 0%, 20%, 40%, 50%, 60%, 80%, and 100%. The engineered Y14 strain could grow robustly using xylose as the sole carbon source, and PPD yield using xylose was higher than that using mixed sugars or glucose (Table 2). Xylose showed some advantages in PPD production, providing another new fermentation strategy for PPD production by *Y. lipolytica*. Moreover, PPD titer using mixed sugars was greater than that using glucose by *S. cerevisiae* [35], indicating that xylose was more suitable for PPD production. Because of different cofactor preferences of XR/XDH, cofactor imbalance may be a limiting factor for PPD production using xylose. In this study, the XR mutation (K270R/N272D) was expressed in *Y. lipolytica*, which may explain higher PPD production using xylose than that using glucose.

**Table 2:** Biomass and metabolites analysis for Y14 with mixed sugars fermentation

Xylose/total sugar	OD_600_	PPD titer (mg/L)^a^	Acetyl-CoA (mmol/L)^a^	Squalene (mg/L)^a^
0	42.09	56.53 ± 1.23	0.178 ± 0.012	3.97 ± 0.21
20%	41.05	68.01 ± 2.38	0.202 ± 0.061	4.28 ± 0.81
40%	39.45	66.12 ± 1.92	0.197 ± 0.013	6.23 ± 1.02
50%	41.53	74.04 ± 2.17	0.203 ± 0.051	7.31 ± 0.15
60%	41.56	72.74 ± 3.28	1.973 ± 0.017	8.29 ± 1.71
80%	41.5	83.23 ± 4.87	1.047 ± 0.082	6.38 ± 0.81
100%	40.35	107.3 ± 1.93	2.352 ± 0.816	10.45 ± 1.38

^a^Each data represents an average ± 1 standard deviation of three parallel fermentations

To investigate the reason higher PPD titer were obtained using xylose than using glucose, metabolites, such as acetyl-CoA and squalene, produced by the Y14 strain at 60 h were analyzed. Acetyl-CoA is an important precursor in the MVA pathway. Cellular acetyl-CoA concentrations may be influenced by carbon sources, contributing to differences in PPD biosynthesis. The concentration of acetyl-CoA produced using xylose was higher than produced using glucose and mixed sugars (Table 2). Furthermore, we analyzed the production of squalene, which is crucial for triterpene biosynthesis via the MVA pathway (Table 2). Higher levels of squalene were produced using xylose as the sole carbon source. In the xylose medium, high acetyl-CoA production resulted in high squalene production compared with that in other media. Thus, PPD titers may be further improved by increasing squalene supply.

### Fermentation by the Y14 strain for PPD production in a 5-L bioreactor

**Fig. 6:**
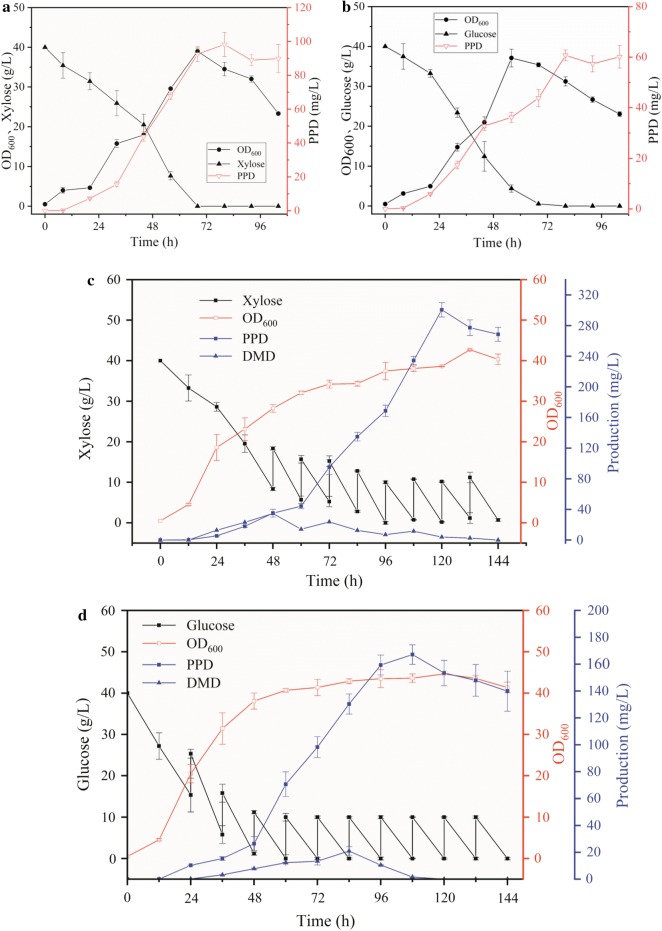
Fermentation of Y14 for PPD production in a 5-L bioreactor. **a** Batch fermentation of engineered strain Y14 in a 5-L bioreactor from xylose. **b** Batch fermentation of engineered strain Y14 in a 5-L bioreactor from glucose. **c** Fed-batch fermentation of engineered strain Y14 in a 5-L bioreactor from xylose. **d** Fed-batch fermentation of engineered strain Y14 in a 5-L bioreactor from glucose. Each data represents an average ± 1 standard deviation of three parallel fermentations

To assess the potential of the Y14 strain as a PPD producer, scale-up experiments were implemented in a 5-L bioreactor using a working volume of 2-L YPD and YPX for cultivating the strain. Xylose and glucose batch fermentations were conducted to test the stability of the engineered strain on fermentation amplification (Fig. 6a, b). Glucose was depleted at 60 h. The highest OD_600_ was 37.08, and PPD titer was 60.72 mg/L. At 72 h, 40 g/L xylose was depleted. OD_600_ was 39.01, and PPD titer was 98.23 mg/L, which was 1.61-times the titer produced using glucose. PPD titer produced by batch fermentation in the 5-L bioreactor was lower than that produced by shake flask fermentation. This may be because the fermentation conditions were better than that in the shake flasks, resulting in faster substrate consumption and insufficient supply of substrate in the later stages. Yang et al. [26] have constructed an engineered strain of *Y. lipolytica* for α-farnesene production and found that the yield in YPD medium by shake flask fermentation could reach 57 mg/L, while that by batch fermentation in 5-L bioreactor could reach 43 mg/L. Sugar consumption was rather fast in the fermenter and could not thus meet the requirements of cell growth and synthesis products. Therefore, for fermentation amplification, we speculated that slower consumption of xylose was more suitable for PPD production than that of glucose.

Fed-batch fermentation in 5-L bioreactor was performed to further increase PPD production (Fig. 6c, d). PPD titer was 300.63 mg/L [yield, 2.505 mg/g (sugar); productivity, 2.505 mg/L/h] using xylose. PPD synthesis quickly entered a stable period using glucose, and the highest yield was 167.17 mg/L [yield, 1.194 mg/g (sugar); productivity, 1.548 mg/L/h]. Similarly, dammarenediol-II (DMD) production was detected at the same time. DMD remained during xylose fermentation, accumulating at 24 h and peaking at 84 h, and then gradually decreased. The difference in the fed-batch fermentation results using xylose and glucose may be due to the quick consumption of glucose, which produced amount of acetyl-CoA, but for the limitations of enzymes activity of MVA pathway, carbon sources flowed to other metabolic branches, causing a reduction of acetyl-CoA used for PPD synthesis. With rapid glucose consumption, cells entered a stable phase. Insufficient supply of other precursors, such as acetyl-CoA and NADPH, resulted in slow PPD production. During xylose-feed fermentation, cells continued to grow slowly, and acetyl-CoA or NADPH were continuously supplied for PPD synthesis, reducing the loss of carbon metabolic flux. In addition, DMD accumulated a little, indicating that the supply of DMD was sufficient to be converted to PPD continuously, producing a greater titer. These results indicate that xylose has great potential for PPD production using acetyl-CoA as a precursor. Studies using *S. cerevisiae* for PPD production using xylose have indicated that xylose fermentation is superior to glucose fermentation in terms of the production of acetyl-CoA. Regulating the metabolic engineering strategy for xylose utilization and product synthesis can enable efficient synthesis of compounds using acetyl-CoA as a precursor and xylose as a substrate.

## Conclusion

This study described a strategy for enhancing PPD production through xylose fermentation with engineered *Y. lipolytica*. *Y. lipolytica* cannot naturally metabolize xylose; however, introduction of heterologous XR/XDH and overexpression of endogenous XKS successfully realized PPD biosynthesis in *Y. lipolytica* using xylose as the sole carbon source. Biomass and PPD production were further increased by overexpression of tHMG1/ERG9/ERG20 in MVA pathway and TAL/TKL/TX in xylose metabolic pathway. Regulation of xylose utilization pathway and product synthesis pathways through metabolic engineering can enable efficient synthesis of terpenoid compounds using xylose as a substrate, which can be a strategy for enhancing terpenoid synthesis in *Y. lipolytica*.

## Materials and methods

### Strains, plasmids, and media

*Yarrowia lipolytica* ATCC 201249 was used as the parental strain for engineering, which was kindly provided by Professor Yingjin Yuan (School of Chemical Engineering and Technology, Tianjin University) [58]. The pINA1269-LUL plasmid was used to amplify the marker *ura3* with the *LoxP* sequence, and the pINA1269-CRE plasmid (Additional file 1: Figure S2) carrying a gene encoding CRE protein was used to recycle the marker *ura3* (codon-optimized sequences were shown in Additional file 1: Table S1). All *Y. lipolytica* strains were cultivated in YPD or YPX medium (2% glucose or 2% xylose, respectively, 2% peptone, and 1% yeast extract) at 30 °C. SC medium (0.67% yeast nitrogen base, 2% glucose, and 2% agar) lacking leucine or uracil was used for screening *Y. lipolytica* transformants. LB medium (1% tryptone, 0.5% yeast extract, and 1% NaCl) supplemented with ampicillin (100 mg/L) was used for culturing *Escherichia coli* DH5α containing recombinant plasmids. Feed solution for fermentation contained 400 g/L xylose or glucose, 20 g/L yeast extract, and 0.5 g/L lysine. The components were sterilized separately and combined in a clean environment.

### Genetic manipulation

Codon-optimized xylose reductase XR (*XYL1*) and XDH (*XYL2*) derived from *S. stipitis* were synthesized by Wuhan Genecreate Biological Engineering Co., Ltd. The optimized nucleotide sequences of the genes are presented in Additional file 1: Table S1. The *DS*, *PPDS*, and *ATR1* genes were synthesized and cloned into pUC57 plasmids by GENEWIZ (Suzhou, China), with codon optimization for *Y. lipolytica*. Endogenous genes encoding XKS (*ylXKS*), xylose transporter (*TX*), transketolase (*TKL*), transaldolase (*TAL*), truncated 3-hydroxy-3-methylglutaryl-coenzyme A reductase (*tHMG1*), farnesyl pyrophosphate synthase (*ERG20*), and squalene synthase (*ERG9*) were amplified using genomic DNA from *Y. lipolytica* ATCC 201249. Promoters and terminators used in the study were amplified from genomic DNA of *Y. lipolytica* ATCC 201249 [51]. All primers used to amplify DNA are listed in Additional file 1: Table S2.

### Strain construction

The expression cassettes used for genome integration were constructed by fusion PCR (Additional file 1: Figure S1). Gene expression cassettes were transformed to *Y. lipolytica* using the LiAc/ssDNA/PEG method, as described previously [52]. The constructed strains are presented in Table 3, and the overall workflow of development of PPD-producing strains was shown Additional file 1: Figure S3.

**Table 3:** Strains used in this work

Strains	Description	Source
Y0	*Yarrowia lipolytica* ATCC 201249: *MATA*, *ura3*-*302*, *leu2*-*270*, *lys8*-*11*, *pex17*-*ha*	[59]
Y1	Y0: *Ku70* deletion::*LUL*	This study
Y2	Y1: *zeta:*:*exp1p*-*XYL1*-*xpr2t*, *gpd1p*-*XYL2*-*lip2t*, *tef1p*-*ylXKS*-*cyc1t*	This study
Y3	Y1: *zeta*::*exp1p*-*ylXYL1*-*xpr2t*, *gpd1p*-*ylXYL2*-*lip2t*, *tef1p*-*ylXKS*-*cyc1t*	This study
Y4	An adaptation of xylose in Y2	This study
Y5	An adaptation of xylose in Y3	This study
Y6	The marker *ura3* was recycled in Y4	This study
Y7	Y6: *rDNA*::*tef1p*-*DS*-*xpr2t*, *exp1p*-*PPDS*-*mig1t*, *gpd1p*-*ATR1*-*lip2t*	This study
Y8	Y6: *rDNA*::*tef1p*-*DS*-*xpr2t*, *exp1p*-*PPDS*-*linker*-*ATR1*-*lip2t*	This study
Y9	The marker *ura3* was recycled in Y8.	This study
Y10	Y9: *POX1*::*Fbainp*-*tHMG1*-*xpr2t*, *gpd1p*-*erg9*-*cyc1t*, *exp1p*-*erg20*-*lip2t*	This study
Y11	The marker *ura3* was recycled in Y10.	This study
Y12	Y11: *POX2*::*exp1p*-*TKL*-*mig1t*, *tef1p*-*TAL*-*lip2t*	This study
Y13	The marker *ura3* was recycled in Y12.	This study
Y14	Y13: *POX3*:: *Pfba1*-*TX*-*cyc1t*	This study

### Adaptation

Cells were cultured in 5 mL SC medium for 7 days. Then, cells were washed using sterile water and transferred to 5 mL SC medium containing 20 g/L xylose (without glucose) for 10 days at 30 °C. Cells were washed again with sterile water and transferred to solid SC medium plates (20 g/L xylose) to culture single colonies, followed by culture in 5 mL selection medium containing xylose (without glucose) to select single colonies. The process was repeated 2–3 times.

### Transcriptional gene expression studies by RT-qPCR

Samples were taken at exponential phase. RNAs was extracted using RNA extraction kit (TIANGEN, China). PrimeScriptTM RT reagent Kit with gDNA Eraser from TaKaRa was used to convent mRNA into cDNA. SYBR^®^ Premix Ex TaqTM II Kit from TaKaRa was used for reverse transcription-quantitative real-time PCR (RT-qPCR). The primers are listed in Additional file 1: Table S3. Triplicate qPCRs were performed. CT (threshold cycle number) values of target genes were normalized using the *erg5* gene as reference. The data obtained were analyzed by applying the 2^−ΔΔCT^ method [59].

### Fermentation in shake flasks

*Yarrowia lipolytica* strains stored at − 80 °C in 25% glycerol were inoculated into 5 mL YPD medium as the seed culture for 24 h at 30 °C while shaking at 220 rpm, and the culture was transferred to 250-mL shake flasks loaded with 50 mL YPD or YPX medium at an initial optical density at 600 nm (OD_600_) of 0.5 under the same cultivation conditions for 4 days. All shake flask fermentation experiments were performed in three parallel experimental groups.

### Batch fermentation

As seed culture for batch fermentation in 5-L bioreactors (Bailun, Shanghai, China), 5 mL preculture was transferred to 150 mL YPD medium in 500-mL shake flasks and cultivated for 24 h at 30 °C while shaking at 220 rpm. The seed medium was inoculated in a 5-L bioreactor at an initial OD_600_ of 0.5 in 2 L YPD or YPX medium. Temperature was maintained at 30 °C, and pH was maintained at 6.0 by adding 20% ammonia water or 3 M H_2_SO_4_. Rotating speed was set at 450 rpm, with an air flow rate of 2 vvm. For fed-batch fermentation in the 5-L bioreactor, 100 mL feed solution was added every 12 h when the sugar was depleted.

### PPD extraction and analysis

PPD was extracted using *n*-butanol, as previously described [35]. The mixture of fermentation broth and *n*-butanol was centrifuged at 11,564×*g* for 10 min, and the *n*-butanol phase was collected for analysis. Samples were analyzed by LC/APCI/MS and quantified by HPLC, as previously reported [60]. Standards were purchased from Meilun Biotechnology Co., Ltd (Dalian, China).

### Analytical methods

Cell growth was determined by measuring the OD_600_ using a UV–VIS spectrophotometer. Concentrations of glucose and xylose were measured using a refractive index detector (Shodex RI-201H) equipped with an Elite P230II pump (Elite Analytical Instruments Co., Ltd., China) and an Aminex HPX-87H column (Bio-Rad, USA). Temperatures of the column and detector were 65 °C and 40 °C, respectively. The mobile phase was 5 mM H_2_SO_4_ at a flow rate of 0.6 mL/min. Acetyl-CoA was analyzed using the Acetyl-CoA ELISA Kit (Shanghai Shuangying Co., Ltd., China). Squalene was analyzed using the Elite HPLC system equipped with an Elite P230II high-pressure pump, UV detection at 203 nm, and the Hypersil C18 column (4.6 mm × 250 mm, 5 µm). Methanol was used as the mobile phase at a flow rate of 1 mL/min.

## Additional file

**Additional file 1.** Additional tables and figures.

## Data Availability

All data generated or analyzed during this study are included in this published article.
