# Supplementary material for: Enhanced protopanaxadiol production from xylose by engineered Yarrowia lipolytica
Source: Microb Cell Fact. 2019 May 18;18:83. doi: 10.1186/s12934-019-1136-7 (PMC6525355; doi:10.1186/s12934-019-1136-7)
Supplement: Supplementary file 1 — Additional file 1. Additional tables and figures. [file 12934_2019_1136_MOESM1_ESM.docx]

**Additional Material**

**Enhanced protopanaxadiol production from xylose by engineered** ***Yarrowia lipolytica***

Yufen Wu ^1, 2,3 a^, Shuo Xu^1, 2, 3 a^, Xiao Gao ^1, 2, 3^, Man Li ^1, 2, 3^, Dashuai Li ^1, 2, 3^, Wenyu Lu^1, 2, 3*^

(1) School of Chemical Engineering and Technology, Tianjin University, Tianjin, People’s Republic of China

(2) Key Laboratory of System Bioengineering (Tianjin University), Ministry of Education, Tianjin, People’s Republic of China

(3) SynBio Research Platform, Collaborative Innovation Center of Chemical Science and Engineering (Tianjin), Tianjin, People’s Republic of China

^a^these authors contributed equally to this work.

^*^**Corresponding author:** Wenyu Lu

**Tel**: +86-022-27892132; **Fax**: +86-022-27892132.

**E-mails**: [wenyulu@tju.edu.cn](mailto:wenyulu@tju.edu.cn)

First auther: Yufen Wu, [yufenwu@tju.edu.cn](mailto:yufenwu@tju.edu.cn)

Order of authers: Shuo Xu, [xushuo@tju.edu.cn](mailto:xushuo@tju.edu.cn)

Xiao Gao, [gaoxiao@tju.edu.cn](mailto:gaoxiao@tju.edu.cn)

Man Li, [18435138418@163.com](mailto:18435138418@163.com)

Dashuai Li, [dashuaili@tju.edu.cn](mailto:dashuaili@tju.edu.cn)

**Tables**

**Table S1. The optimized sequences of *cre*, *XYL1*, and *XYL2*.**

| **Codon optimized sequences of *cre*** |
| --- |
| ATGTCCAACCTGCTGACCGTCCACCAGAACCTGCCCGCCCTGCCCGTCGATGCCACCTCTGACGAGGTCCGAAAGAACCTGATGGACATGTTCCGAGACCGACAGGCCTTCTCCGAGCACACCTGGAAGATGCTGCTGTCCGTCTGCCGATCCTGGGCCGCTTGGTGCAAGCTGAACAACCGAAAGTGGTTCCCCGCCGAGCCCGAGGACGTTCGAGACTACCTGCTGTACCTGCAGGCCCGAGGCCTGGCCGTCAAGACCATCCAGCAGCACCTGGGCCAGCTGAACATGCTGCACCGACGATCCGGCCTCCCCCGACCCTCTGACTCTAACGCCGTCTCCCTCGTCATGCGACGAATTCGAAAGGAGAACGTCGATGCCGGCGAGCGAGCCAAGCAGGCCCTCGCTTTCGAGCGAACCGACTTCGACCAGGTTCGATCCCTGATGGAGAACTCCGACCGATGTCAGGACATCCGAAACCTGGCCTTCCTGGGCATCGCCTACAACACCCTGCTGCGAATCGCCGAGATTGCCCGAATTCGAGTGAAGGACATCTCCCGAACCGACGGTGGCCGAATGCTGATCCACATCGGTCGAACCAAGACCCTCGTGTCCACCGCCGGAGTCGAGAAGGCCCTGTCTCTGGGCGTCACCAAGCTGGTGGAGCGATGGATCTCCGTCTCCGGAGTGGCCGACGACCCCAACAACTACCTGTTCTGCCGAGTCCGAAAGAACGGTGTCGCCGCCCCCTCTGCCACCTCTCAGCTGTCCACCCGAGCCCTGGAGGGCATCTTCGAGGCCACCCACCGACTGATCTACGGCGCCAAGGACGACTCTGGCCAGCGATACCTGGCCTGGTCCGGTCACTCCGCCCGAGTTGGCGCCGCTCGAGACATGGCCCGAGCTGGTGTTTCCATTCCCGAGATCATGCAGGCCGGCGGTTGGACCAACGTCAACATCGTCATGAACTACATCCGAACCCTCGACTCCGAGACCGGTGCCATGGTCCGACTGCTGGAGGACGGTGACTAA |
| **Codon optimized sequences of *XYL1*** |
| ATGCCCTCTATCAAGCTGAACTCTGGCTACGACATGCCCGCCGTCGGCTTCGGCTGCTGGAAGGTGGACGTGGACACCTGTTCTGAGCAGATCTACCGAGCCATCAAGACCGGCTACCGACTGTTCGACGGCGCCGAGGACTACGCCAACGAGAAGCTGGTCGGAGCCGGCGTGAAGAAGGCCATCGACGAGGGCATCGTGAAGCGAGAGGACCTGTTCCTGACCTCTAAGCTGTGGAACAACTACCACCATCCTGACAACGTCGAGAAGGCCCTGAACCGAACTCTGTCTGACCTCCAGGTGGACTACGTGGACCTGTTTCTCATTCACTTCCCCGTGACCTTCAAGTTCGTGCCCCTGGAAGAGAAGTACCCTCCTGGCTTCTACTGCGGCAAGGGCGACAACTTCGACTACGAGGACGTGCCCATCCTCGAGACTTGGAAGGCCCTCGAGAAGCTCGTGAAGGCCGGCAAGATCCGATCTATCGGCGTGTCTAACTTCCCCGGTGCTCTGCTGCTGGACCTGCTGCGAGGCGCCACCATCAAGCCCTCTGTGCTGCAGGTCGAGCATCACCCCTACTTGCAGCAGCCCCGACTGATCGAGTTCGCTCAGTCCCGAGGAATCGCCGTGACCGCCTACTCTTCGTTCGGACCCCAGTCTTTCGTCGAGCTGAACCAGGGACGAGCCCTGAACACCTCGCCTCTGTTCGAGAACGAGACTATCAAGGCCATTGCCGCCAAGCACGGCAAGTCTCCCGCTCAGGTGCTGCTCCGATGGTCCTCTCAGCGAGGCATTGCCATCATTCCCCGATCTGACACCGTGCCTCGACTGCTCGAGAACAAGGACGTGAACTCTTTCGACCTGGACGAGCAGGACTTCGCCGACATTGCCAAGCTGGACATCAACCTGCGATTCAACGACCCCTGGGACTGGACAAGATCCCCATCTTCGTGTAA |
| **Codon optimized sequences of *XYL2*** |
| ATGACTGCTAACCCCTCTCTGGTGCTGAACAAGATCGACGACATCTCTTTCGAGACTTACGACGCTCCCGAGATCTCTGAGCCCACCGACGTGCTGGTGCAGGTCAAGAAGACCGGCATCTGCGGCTCTGACATCCACTTCTACGCCCACGGCCGAATCGGCAACTTCGTGCTGACCAAGCCTATGGTGCTGGGCCACGAGTCTGCCGGCACCGTGGTCCAGGTCGGCAAGGGCGTGACCTCTCTGAAGGTGGGCGACAACGTGGCTATCGAGCCCGGCATTCCCTCTCGATTCTCTGACGAGTACAAGTCTGGCCACTACAACCTGTGTCCTCACATGGCCTTCGCCGCTACTCCCAACTCTAAGGAAGGCGAGCCCAACCCTCCTGGCACTCTGTGCAAGTACTTCAAGTCTCCCGAGGACTTCCTGGTGAAGCTGCCCGACCATGTGTCTCTCGAGCTGGGCGCCCTGGTCGAGCCCCTGTCTGTGGGAGTGCACGCCTCTAAGCTGGGCTCTGTGGCCTTCGGAGACTACGTGGCCGTGTTCGGCGCTGGCCCCGTGGGCCTGCTGGCCGCTGCCGTGGCCAAGACCTTCGGCGCCAAGGGCGTCATCGTGGTGGACATCTTCGACAACAAGCTGAAGATGGCCAAGGACATCGGCGCTGCTACCCACACCTTCAACTCCAAGACCGGCGGATCTGAGGAACTGATCAAGGCTTTCGGCGGCAACGTGCCCAACGTGGTGCTCGAGTGCACCGGCGCTGAGCCCTGCATCAAGCTCGGCGTGGACGCTATCGCTCCCGGCGGACGATTCGTGCAGGTCGGAAACGCCGCTGGACCCGTGTCTTTCCCCATCACCGTGTTCGCCATGAAGGAACTGACCCTGTTCGGCTCTTTCCGATACGGCTTCAACGACTACAAGACCGCCGTGGGCATTTTCGACACCAACTACCAGAACGGCCGAGAGAACGCTCCCATCGACTTCGAGCAGCTGATCACCCACCGATACAAGTTCAAGGACGCCATCGAGGCCTACGACCTGGTGCGAGCCGGAAAGGGCGCCGTGAAGTGCCTGATCGACGGCCCCGAGTAA |

**Table S2. Primers used in this study.**

| **Name** | **Primer sequence (5’-3’)** |
| --- | --- |
| Kup-F | GGAATGGATTTCACATCTGGAGAACGA |
| Kup-R | TCATTCGTCGTGGGGGTATCG |
| Kup-LUL-F | CGAATCATCTATATCACAGACGACGATACCCCCACGACGAATGAGTTGTAAAACGACGGCCAGTG |
| LUL-R-Kdown | GTATCTTTGCGACTCAAATCCGCCAGGCATGCGTAGACGAGTGAAGCACGTGATGAATTCGAGCTC |
| Kdown-F-LUL | TTCACTCGTCTACGCATGCCT |
| Kdown-R | TCACTTCCCATAGTACTTTTTGACCAC |
| Check-F | GGAATGGATTTCACATCTGGAGAACGA |
| Check-R | GCCTCGTTCAGAATGACACGTATAGAA |
| Zetaup-F | AGAGAATCGGCGTTACCTCTCTCACA |
| Zetaup-R-Pexp | GGCGCCAAACTCAGTGCTTTGTGCGTACCAGGG |
| Zetaup-Pexp-F | TGGTACGCACAAAGCACTGAGTTTGGCGCCCGTTTTTTC |
| Pexp-R-XYL1 | TTCAGCTTGATAGAGGGCATTGCTGTAGATATGTCTTGTGT |
| Pexp-XYL1-F | ATATCTACAGCAATGCCCTCTATCAAGCTGAAC |
| XYL1-R-XPR2t | CAACGTGGGGACAGGTTACACGAAGATGGGGATCTT |
| XYL1-XPR2t-F | CCCATCTTCGTGTAACCTGTCCCCACGTTGCCGGTC |
| XPR2t-R-Pgpd1 | GGACATCCTACTGCGTCGGACACGGGCATCTCACTT |
| XPR2t-Pgpd1-F | GATGCCCGTGTCCGACGCAGTAGGATGTCCTGCACG |
| Pgpd1-R-XYL2 | GGGGTTAGCAGTCATTGTTGATGTGTGTTTAATTCA |
| Pgpd1-XYL2-F | AAACACACATCAACAATGACTGCTAACCCCTCTCTG |
| XYL2-R-Lip2t | AGAGTGATAAATAGCTTACTCGGGGCCGTCGATCAG |
| XYL2-Lip2t-F | GACGGCCCCGAGTAAGCTATTTATCACTCTTTACAA |
| Lip2t-R-tef1p | CAACCCGGTCTCTTCCACCTGTGTCAATCTTCTC |
| Lip2t-tef1p-F | AGAAGATTGACACAGGTGGAAGAGACCGGGTTGGCGGCGTA |
| tef1p-R-ylXKS | TTTGAATGATTCTTATACTCAGAAGGAAAT |
| tef1p-ylXKS-F | ATTTCCTTCTGAGTATAAGAATCATTCAAAATGTATCTCGGACTGGATCTT |
| ylXKS-R-cyc1t | AACTAATTACATGATTTATTTCTCCAGGCAGGCGTT |
| ylXKS-cyc1t-F | TGCCTGGAGAAATAAATCATGTAATTAGTTATGTCA |
| cyc1t -R -Leu2 | GACGGAATTCGCAAATTAAAGCCTTCGAGC |
| cyc1t-Leu2-F | CTTTAATTTGCGAATTCCGTCGTCGCCTGAG |
| Leu2-R-Zetad | GAGTGTTACAAAGAATTCATGTCACACAAACCG |
| Leu2-Zetad-F | CATGAATTCTTTGTAACACTCGCTCTGGAGAGTTAGTCAT |
| Zetad-R | CTGCAGTCGTAAGACCCAGGTGGTGTGT |
| Pexp--R-ylXR | GAGCTTGAAGGACATAGAGCTGGGTTAGTTTGTGTA |
| Pexp-ylXR-F | ACAAACTAACCCAGCTCTATGTCCTTCAAGCTCGCCTCC |
| ylXR-R-xpr2t | TGGGGACAGGTTAGGCGAAAATGGGAAGGTTAGCGTAGAC |
| ylXR-xpr2t-F | CCTTCCCATTTTCGCCTAACCTGTCCCCACGTTGCCGGTC |
| Pgpd1-R-ylXDH | GAACAAATGACGGGTTAGAAGACATTGTTGATGTGTGTTTAATTCA |
| Pgpd1-ylXDH-F | AAACACACATCAACAATGTCTTCTAACCCGTCATTT |
| ylXDH-R-Lip2t | GAGTGATAAATAGCCTACTCCTCCTCGGGACCGTC |
| ylXDH-Lip2t-F | GGTCCCGAGGAGGAGTAGGCTATTTATCACTCTTTACAA |
| rDNAup-F | TCGATCCTAAGGGGTGGCATAACTGTCGC |
| rDNAup-R-Ptef1 | ACGCCGCCAACCCGGTCTCTTGGCTACCTTAAGAGAGTCAT |
| Ptef1-F-rDNAup | ATGACTCTCTTAAGGTAGCCAAGAGACCGGGTTGGCGGCGTA |
| Ptef1-R-DS | GCGACCTTCAGCTTCCACATTTTGAATGATTCTTATACTCA |
| DS-F- Ptef1 | GAGTATAAGAATCATTCAAAATGTGGAAGCTGAAGGTCGCC |
| DS-R-xpr2t | ACCGGCAACGTGGGGACAGGTTAGATCTTCAGCTGCTGGTG |
| xpr2t -F-DS | ACCAGCAGCTGAAGATCTAACCTGTCCCCACGTTGCCGGTC |
| xpr2t -R-Pexp1 | TCACCGAAACGTGTGGGGCTCGAAAAAACGGGCGCCAAACTCTCGGACACGGGCATCTCACTTGC |
| Pexp1-F-xpr2t | TATTTTTATTTTCCATACATACGCAAGTGAGATGCCCGTGTCCGAGAGTTTGGCGCCCGTTTTTTC |
| Pexp1-R-PPDS | AGGGAGAAGAACAGGACCATTGCTGTAGATATGTCTTGTGT |
| PPDS-F-Pexp1 | CACAAGACATATCTACAGCAATGGTCCTGTTCTTCTCCCTG |
| PPDS-R-mig1t | TAAATTATCGACCGGCCAGTGTTAGTTGTGAGGGTGCAGATG |
| mig1t -F-PPDS | TCTGCACCCTCACAACTAACACTGGCCGGTCGATAATTTA |
| mig1t -R-Pgpd1 | TCCACACCCCACAAAAAGACCCGTGCAGGACATCCTACTGCGAAACCCAAAAGGGCCGAAGG |
| Pgpd1-F-mig1t | TCCATCTCAGAGCCTCGGCCCAGCCTTCGGCCCTTTTGGGTTTCGCAGTAGGATGTCCTGCAC |
| Pgpd1-R-ATR1 | GCGTACAGAGCGGAGGTCATTGTTGATGTGTGTTTAATTCA |
| ATR1-F-Pgpd1 | GAATTAAACACACATCAACAATGACCTCCGCTCTGTACGCT |
| ATR1-R-lip2t | TGTAAAGAGTGATAAATAGCTTACCAGACGTCTCGCAGGTA |
| lip2t -F-ATR1 | TACCTGCGAGACGTCTGGTAAGCTATTTATCACTCTTTACAA |
| lip2t-R-LUL | ACTGGCCGTCGTTTTACAACTCCACCTGTGTCAATCTTCTC |
| lip2t-LUL-F | ATTGACACAGGTGGAGTTGTAAAACGACGGCCAGTG |
| LUL-R-rDNAdown | TAATTAGATGACGAGGCATTGCACGTGATGAATTCGAGCTC |
| LUL-rDNAdown-F | CTCGAATTCATCACGTGCAATGCCTCGTCATCTAATTAG |
| rDNAdown-R | CTTCGGTATGATAGGAAGAGCCGACATCG |
| Ptef1-R-DS | GCGACCTTCAGCTTCCACATTTTGAATGATTCTTATACTCA |
| DS-F- Ptef1 | GAGTATAAGAATCATTCAAAATGTGGAAGCTGAAGGTCGCC |
| DS-R-xpr2t | ACCGGCAACGTGGGGACAGGTTAGATCTTCAGCTGCTGGTG |
| xpr2t -F-DS | ACCAGCAGCTGAAGATCTAACCTGTCCCCACGTTGCCGGTC |
| xpr2t -R-Pexp1 | TCACCGAAACGTGTGGGGCTCGAAAAAACGGGCGCCAAACTCTCGGACACGGGCATCTCACTTGC |
| Pexp1-F-xpr2t | TATTTTTATTTTCCATACATACGCAAGTGAGATGCCCGTGTCCGAGAGTTTGGCGCCCGTTTTTTC |
| Pexp1-R-PPDS | AGGGAGAAGAACAGGACCATTGCTGTAGATATGTCTTGTGT |
| PPDS-F- Pexp1 | CACAAGACATATCTACAGCAATGGTCCTGTTCTTCTCCCTG |
| PPDS-linker-R | GTCTTCTTCCAACCGGAGGAGGTGGAACCGTTGTGAGGGTGCAGATGGAT |
| ATR1-R-lip2t | TGTAAAGAGTGATAAATAGCTTACCAGACGTCTCGCAGGTA |
| lip2t -F-ATR1 | TACCTGCGAGACGTCTGGTAAGCTATTTATCACTCTTTACAA |
| POX1A-F | TGAGTATAATCCGTCTCCGTTCCCACT |
| POX1A-R-Pfbain | TACTGCGTACACTGTTATCGGTGAATGTGTTGGTTGTCATTGT |
| POX1A-Pfbain-F | AACCAACACATTCACCGATAACAGTGTACGCAGTACTATAGAGGAA |
| Pfbain-R-tHMG1 | AATGACGATAGGCATAGAGCTGGGTTAGTTTGTGTAGAGAGT |
| Pfbain-tHMG1-F | ACTCTCTACACAAACTAACCCAGCTCTATGCCTATCGTCATTGAGAAG |
| tHMG1-R-xpr2t | AACGTGGGGACAGGCTATGACCGTATGCAAATATTCGAACC |
| tHMG1-xpr2t-F | TTCGAATATTTGCATACGGTCATAGCCTGTCCCCACGTTGCCGGTC |
| xpr2t-R-Pexp1 | TTCCATCTCCAAGCACTCGGACACGGGCATCTCACTTGCGTAT |
| xpr2t- Pexp1-F | TGAGATGCCCGTGTCCGAGTGCTTGGAGATGGAAGCCGGTAGAAC |
| Pexp1-R-erg9 | CAAGAGCAGTTCGATGAGTTTTCCCATTGTTGATGTGTGTTTAATTCAAG |
| Pexp1-erg9-F | TTGAATTAAACACACATCAACAATGGGAAAACTCATCGAACTGCT |
| erg9-R-lip2t | GTAAGCGTGACATAACTAATTACATGATCTAATCTCTCAGAGGAAACATCT |
| erg9-lip2t-F | AAGATGTTTCCTCTGAGAGATTAGATCATGTAATTAGTTATGTCACG |
| lip2t-R-Pgpd1 | TCTCCAATAATTATAATTAGTTGCCGGTCAGCTGGGTTCTTCT |
| Lip2t- Pgpd1-F | CAAATTAAAGCCTTAACACATTCTATGCCTATCGGTTA |
| Pgpd1-R-erg20 | AATTTCGCCTTGGACATTGTTGATGTGTGTTTAATTCAAG |
| Pgpd1-erg20-F | TTCTTGAATTAAACACACATCAACAATGTCCAAGGCGAAATTCGAAAG |
| erg20-R-cyc1t | ACATAACTAATTACATGATCTACTTCTGTCGCTTGTAAATCT |
| erg20-cyc1t-F | CAAGATTTACAAGCGACAGTAATTAAGATCATGTAATTAGTTATGTCACGCTTA |
| cyc1t-R-LUL | GTTTTACAACGCAAATTAAAGCCTTCGAGCGTCCCAA |
| cyc1t-LUL-F | GTTTTACAACGCAAATTAAAGCCTTCGAGCGTCCCAA |
| LUL-R-POX1B | AATGTATCTCATAATTACTAACAGCACGTGATGAATTCGAGCTCGGTACC |
| LUL-POX1B-F | CTCGAATTCATCACGTGCTGTTAGTAATTATGAGATACATTCTTT |
| POX1B-R | ATTGTTGACCTGGCTCGTTTTGTGCCTCTG |
| POX2A-F | ACGTGCAACCAAATGTCACTGT |
| POX2A-R-Pexp1 | GTTTTGGTACACGAGATAGAAGGGGGTTTT |
| POX2A-Pexp1-F | CCTTCTATCTCGTGTACCAAAACCCCGTTTTTTCGAGCCCCACACGT |
| Pexp1-R-TKL | AAAATTGGGGAGCCATTGCTGTAGATATGTCTTGTGTGTAAGG |
| Pexp1-TKL-F | CACAAGACATATCTACAGCAATGGCTCCCCAATTTTCAAAGACTGAC |
| TKL-R-mig1t | TTATCGACCGGCCAGTGTTAGACACCGTGGCCGGGTCGGGT |
| TKL-mig1t-F | CCGACCCGGCCACGGTGTCTAACACTGGCCGGTCGATAATTTAACG |
| mig1t-R-Ptef1 | CGCCAACCCGGTCTCTGAAACCCAAAAGGGCCGAAG |
| mig1t- Ptef1-F | GCCCAGCCTTCGGCCCTTTTGGGTTTCAGAGACCGGGTTGGCGGCGTA |
| Ptef1-R-TAL | GTTCAAGAGAGTTGGAAGACATTTTGAATGATTCTTATACTCAGAAGGA |
| Ptef1-TAL-F | CTGAGTATAAGAATCATTCAAAATGTCTTCCAACTCTCTTGAACAGCTT |
| TAL-R-lip2t | CTAAGCGGAGAGCTTGGTCTCAATGAGCTT |
| TAL-lip2t-F | GAGACCAAGCTCTCCGCTTAGGCTATTTATCACTCTTTACAACTTCTAC |
| lip2t-R | GCACTGGCCGTCGTTTTACAACTCCACCTGTGTCAATCTTCTCAAGCTG |
| lip2t-LUL-F | CTTGAGAAGATTGACACAGGTGGAGTTGTAAAACGACGGCCAGTGC |
| LUL-R | AATATATCCTTCAATCCATGCACGTGATGAATTCGAGCTCGGTACC |
| LUL-POX2B-F | GAGCTCGAATTCATCACGTGCATGGATTGAAGGATATATTGTTACTAA |
| POX2B-R | GGTAGCCGACATATACTGTAAGCATCTGCGCCTTG |
| POX3A-F | CGGTAAAAATAGACCAATCAGAA |
| POX3A-R-Pfba | TGTGTGTATCGTAGAGGTAGTGA |
| POX3A-Pfba1-F | GTCACTACCTCTACGATACACACAAACAGTGTACGCAGTACTATAGA |
| Pfba1-R-TX | TGTGTGATGTGTAGTTTAGATTTCGAATCTGTGGGGAAAG |
| Pfba1-TX-F | CGAAATCTAAACTACACATCACACAATGTCGCACCGGCCCTGGGATTG |
| TX-R-cyc1t | TCACCTATCAGCATTTTCACCCATTTCCAGAGTCTCGGTG |
| TX-cyc1t-F | GAAAATGCTGATAGGTGAATCATGTAATTAGTTATGTCACG |
| LUL-POX3B-F | CTCGAATTCATCACGTGCTAGATGGAGCGTGTGTTCTGAGT |
| POX3B-R | GCTCATTTTCGGTCTCCAAACTG |

**Table S3. Primers used for RT-qPCR.**

| **Name** | **Primer sequence (5’-3’)** |
| --- | --- |
| XYL1-F | GGTGCTGCTCCGATGGTC |
| XYL1-R | GGTCGTTGAATCGCAGGTT |
| XYL2-F | GCGGCTCTGACATCCACTT |
| XYL2-R | TGGGCTCGCCTTCCTTAG |
| ylXKS-F | CAGTATCAAAATGAGACTCCAACG |
| ylXKS-R | TTCTTGTATTTGACCTCTTCTTCG |
| erg5-F | CCAACAGAGCCCTCAGCAT |
| erg5-R | GCGGCAGTCACCAGGTAGTA |

**Figures**


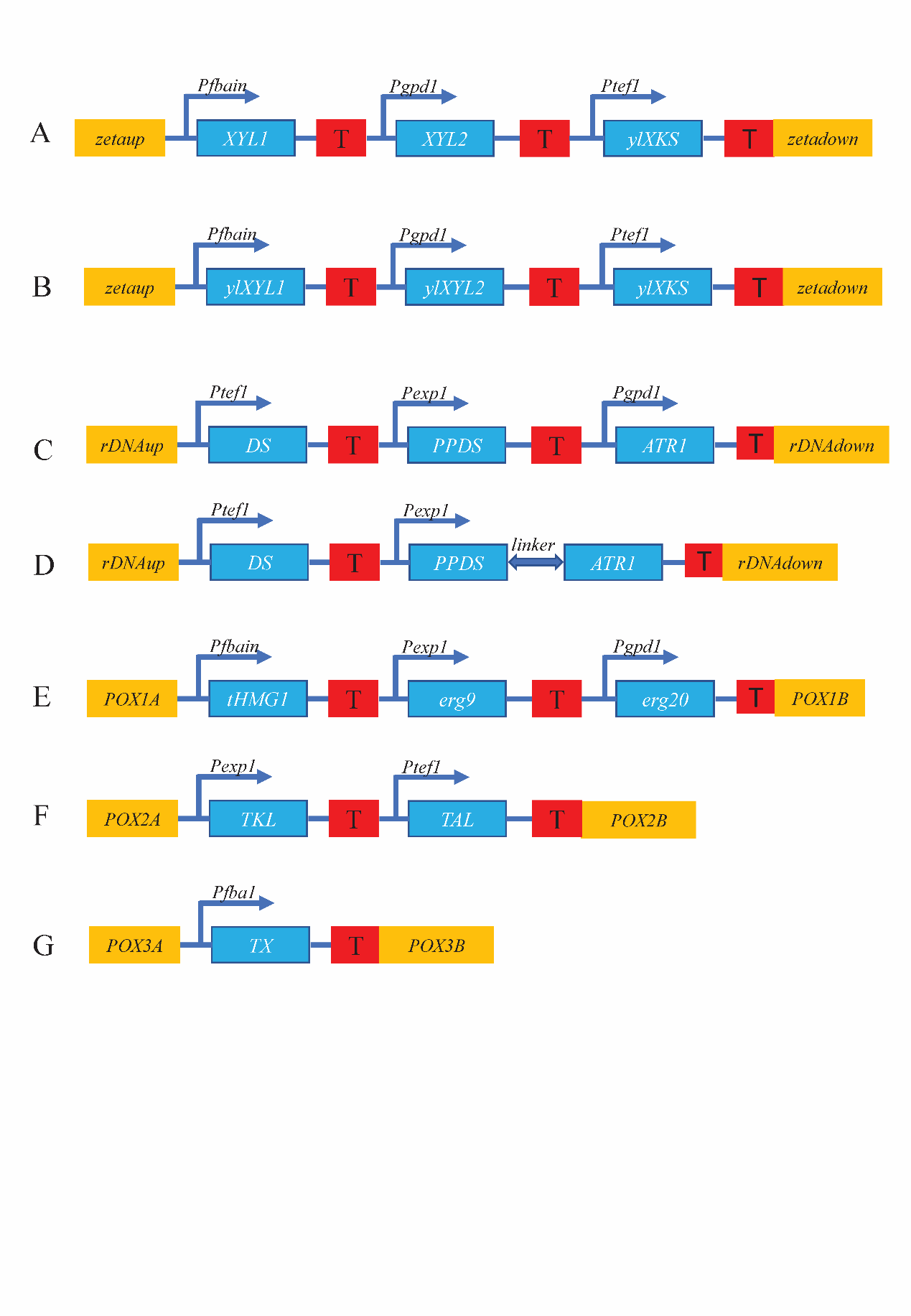


**Figure S1. The construction of expression modules in this study.**


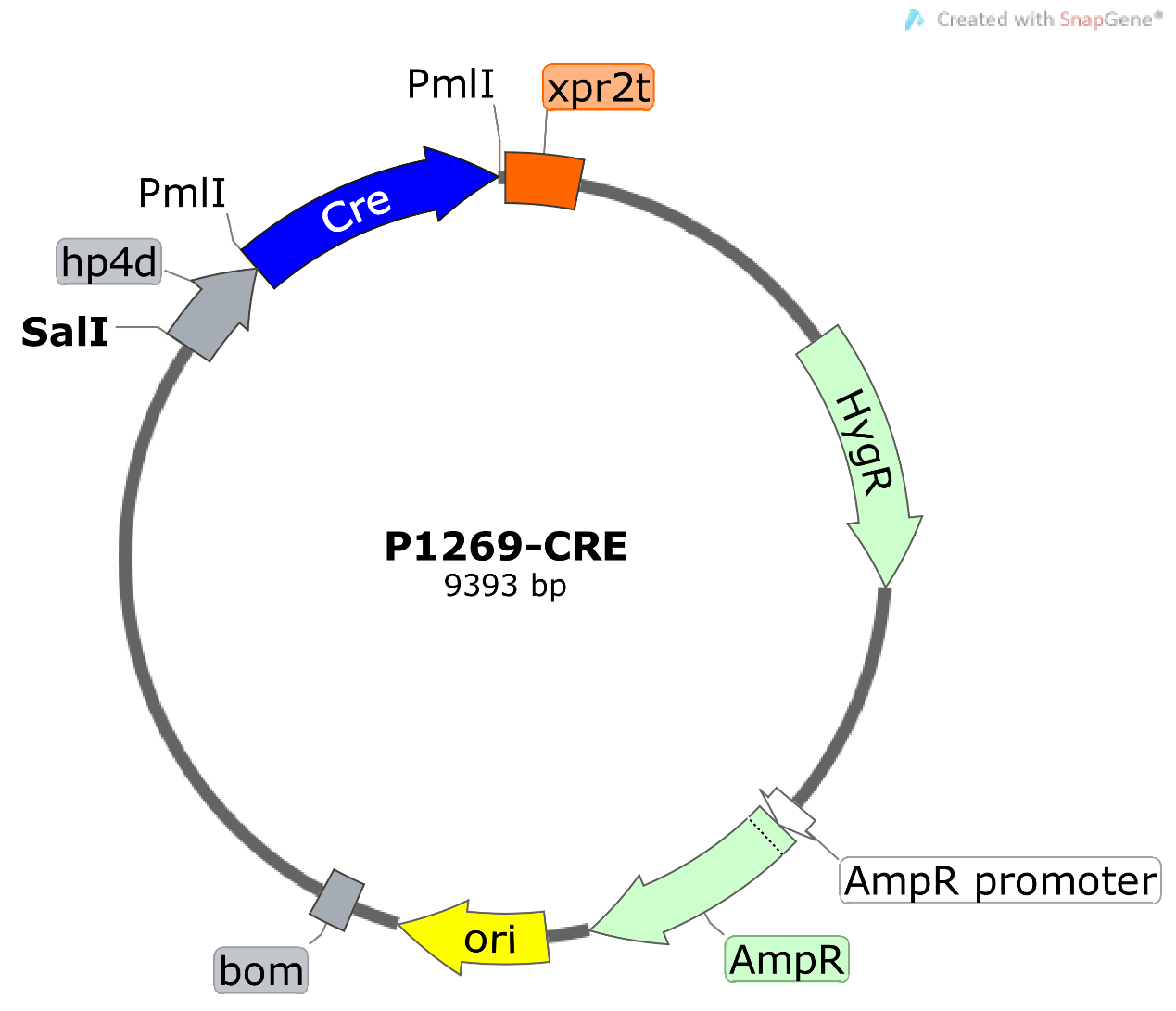


**Figure S2. The diagram of pINA1269-CRE.**


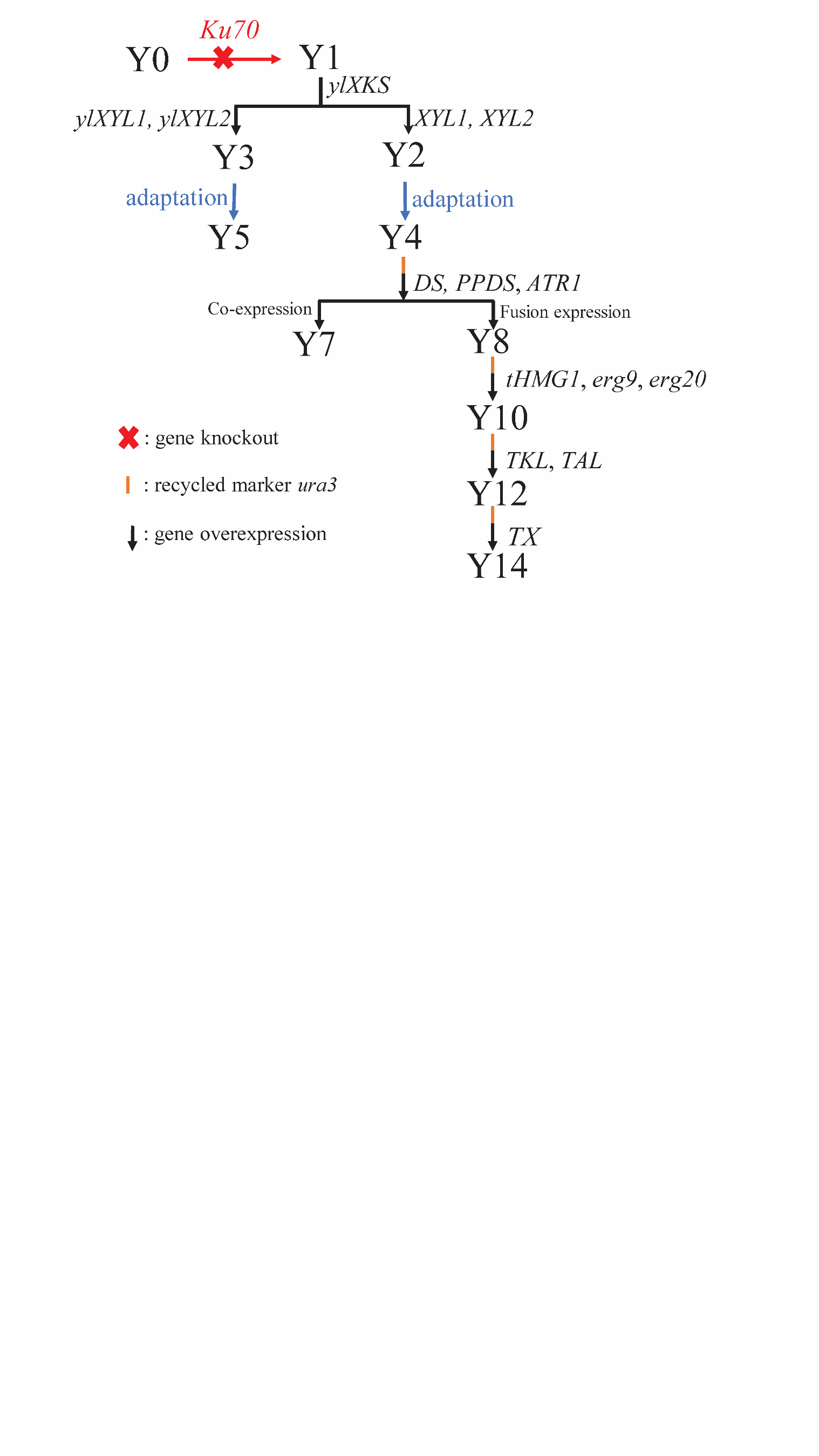


**Figure S3. The overall workflow of development of PPD-producing strains.**
